# Dermatology

**Published:** 1905-08-05

**Authors:** 


*(To be continued.)*

### DERMATOLOGY.

**Seborrhœa and Psoriasis.**—A valuable contribution towards settling the vexed subject of the connection between seborrhœa, seborrhoic dermatitis, and psoriasis is made by Brimacombe,[1] who discusses the histopathological features and the relationship between these affections. Taking seborrhœa to mean what Sabouraud meant by it—"Flux of Sebum," he states that in this condition there is a hypertrophy of the sebaceous glands. In seborrhoic dermatitis, however, there is a diminution of activity, and sometimes an atrophy of the sebaceous glands, with an increase in size and function of the coil glands, the secretion being mainly sudoriparous. Owing to the increase of vascularity the epithelial layers are increased, the increase being due to a swelling of the cells and a dilatation of the lymph spaces between them. At places the epidermis is narrowed and leucocytes are found in the cutis, the corneous cells being heaped up round them like a roof. In this the appearance suggests an incipient psoriasis. The presence of organisms he considers to bear no causal relationship to the disease, but to be due to coincidence, for such germs are found in the superficial layers of the skin only and not in the deeper layers. The increase in the sweat glands is compensatory for the sebaceous glands which have lost their function, fat being found in normal sweat. In psoriasis itself the same conditions exist, only there is an atrophy of the coil glands as well as of the sebaceous glands. The natural secretions of the skin failing, the accompanying inflammation must be looked upon as a secondary process, and not, as stated by Unna and Elliott, "to be a primary affection and not a disturbance in secretion."

**Cancer, Leucodern, and Sclerodern.**—Lenthal Cheatle[2] attempts to compare the points of incidence in cancer, leucoderma, and scleroderma. He has previously drawn attention to the fact that rodent ulcers occur on the trunk at points at which the nerves become cutaneous. With the material collected, including 138 cases supplied by Dr. Sequeira, he is unable to demonstrate that all occur on similar spots on the face, but states that they appear most commonly on points at which nerves become cutaneous, notably the infra-orbital, infra-trochlear, temporo-malar, and lachrymal. According to Dr. Head, where only one skin area of a nerve is affected by a disease, the cause is more likely to be a local one than if there are more than one skin area affected, when the cause is probably central. The points of incidence in rodent ulcer on the face and trunk are common to the points of incidence of the so-called neurotrophic diseases leucoderma and scleroderma. Hence it appears that the points of incidence of these diseases are where the nerves become cutaneous. In these there is, as a rule, only one maximum point of a nerve affected, and so, according to Dr. Head, the neurotrophic factor is probably peripheral. Cheatle publishes a number of photographs of collected cases, and deduces from his observations of these cases that the incidence of cancer may be due to the direct or indirect nervous influence of the area in which it occurs, as also he believes to be the case in leucoderma and scleroderma. If this is so, then the tenability of part or all of the cleft-inclusion theory is threatened. And, in addition, the incidence of rodent ulcers on the trunk is not usually on the position of clefts as these are known. Rodents are formed only on one cutaneous maximum point and not on two or more, and hence, according to Head, the cause is either not central, or is of peripheral modification of central nerve influence, and not only segmental. Also he cannot find evidence that the points selected by rodent-ulcer are the selected sites of infective diseases, and he considers that these points tend to militate against the parasitic theory of cancer.

**Angio-Neurotic Œdema.**—The association of gastro-intestinal symptoms, acute in character but of transient duration, occurring in a patient the subject of angio-neurotic œdema has been noticed by several observers. The causation of the pain, etc., has up till now been only a matter of conjecture. A case has recently passed through the hands of Morris,[3] who had the good fortune to obtain a portion of the gastric mucous membrane in a state of acute œdema during one of these gastro-intestinal attacks. The patient, a male aged 21, gave a history of frequent attacks of transient œdema of various parts of the body, including dangerous conditions of œdema of the glottis. During observation in hospital a complete record of his case was made, and an Ewald test breakfast given of the acidity and peptic activity of the stomach estimated. At this time the stomach mucosa is supposed to have been wounded by the stomach tube, for at a subsequent lavage a fairly large portion of the gastric mucosa was removed by the tube, which was subjected to microscopical investigation. The acidity was also found to have decreased considerably. The microscopical findings were those of acute œdema, which was chiefly confined to the intestinal tissue, and "non-inflammatory in origin." Morris considers that the pain may be due to a stretching or pressure upon the nerve-endings, especially as there was evidence of the œdematous fluid being stored at considerable pressure. "To conclude," he says, "that all attacks of gastric disturbance met with in acute circumscribed œdema are attributable to local œdema of the stomach wall would be unwarrantable. To assume that a simple non-inflammatory œdema is the only pathologico-anatomical alteration which may occur in this disease would be equally unjustifiable." That simple œdema of the stomach wall does occur concomitantly with attacks of nausea, vomiting, and epigastric pain at times, and that there is probability of such œdema lying at the base of all gastro-intestinal symptoms in angio-neurotic œdema is greatly strengthened by this report.

**Acne Rosacea.**—In acne rosacea Lloyd[4] has found that great and in some cases immediate, benefit has followed slight intranasal operation. After pressure on the septum by polypi and enlarged turbinals has been relieved or hypertrophic rhinitis treated, not only the nose has improved, but the redness of the face has improved also, in some cases within a week after the operation.

**Lupus Erythematosus.**—The treatment of lupus erythematosus by means of quinine internally and iodine externally, as suggested by Holländer, consisted in the primary determination of any possible idiosyncrasy by the administration of ·05 gramme (= 1 grain) of sulphate or chloride of quinine, then the repetition of this dose thrice daily, while the patches were painted with several coats of tincture of iodine. The painting was carried out morning and evening afterwards for five or six days at a time, with intervals of the same length. White patches of sound epidermis remained after separation of the crust, and occasionally the affected areas were observed to swell with a circumscribed reddened area as with a tuberculin reaction. Moritz Oppenheim[5] has modified this method of Holländer's. After establishing the presence of freedom from any idiosyncrasy by means of a dose of ·05 gramme of sulphate or bisulphate of quinine, then for three days, morning and evening, 0·50 gramme (8 grains circ.) quinine are administered, the patches are cleansed with alcohol and ether and iodine painted on. Every third day of the administration 0·5 gramme quinine is added till the dose is increased to eight half-grammes (61 grains) per diem according to the intensity of the case. At this dose the patient remains until the foci of the disease are removed, when the daily dose is diminished 0·50 gramme every third day till the dose reached is only 0·5 gramme daily, when the treatment ceases. The treatment is not intermitted except for noises in the ear or deafness, but immediately on the cessation of these the original dose is recommenced and correspondingly increased. No untoward symptoms follow these large doses of quinine. All the patients treated were taking over 100 grammes quinine each week, and in none did there appear any objectionable after-results and the patients felt no ill effect. The gradual increase of the dose had the result of accustoming the patient to the larger dose of a drug the more rapid increase of which would not be tolerated. When a longer period than three or four days between the increments was used the patches ceased to show improvement. On increasing the dose after this reddening and itching would follow before further improvement. The doses must not be diminished too soon either, as if a complete cure is not effected it is not in every case that a good result occurs on a second attempt. His conclusions are as follows:—(1) The treatment should be tried in all chronic cases; (2) In no cases was there a negative result; (3) In the absence of idiosyncrasy large amounts of quinine could be taken when the dose was increased gradually; (4) The inverse method of giving iodide of sodium internally and a solution of quinine externally had no effect.

[1] Lancet, Feb. 18, 1905. [2] Brit. Med. Jour., April 29, 1905. [3] Amer. Jour. Med. Sci, Nov. 1904. [4] Brit. Med. Jour., Jan. 14, 1905. [5] Weiner Klin. Woch., Jan. 19, 1905.

